# Influence of selenium supplementation on carbohydrate metabolism and oxidative stress in pregnant women with gestational diabetes mellitus

**DOI:** 10.2478/jomb-2019-0034

**Published:** 2020-01-23

**Authors:** Hadjer Saifi, Yassine Mabrouk, Rayane Saifi, Messaouda Benabdelkader, Mouldi Saidi

**Affiliations:** 1 National Center of Nuclear Science and Technology (CNSTN), Laboratory of Biotechnology and Nuclear Technology, Sidi Thabet Technopark, Ariana, Tunisia + University of Tunis El Manar, Faculty of Mathematical, Physical and Natural Sciences of Tunis, Department of Biological Sciences, Tunis, Tunisia; 2 National Center of Nuclear Science and Technology (CNSTN), Laboratory of Biotechnology and Nuclear Technology, Sidi Thabet Technopark, Ariana, Tunisia; 3 University of Mohamed Khider, Department of Agricultural Sciences, Laboratory of Ecosystems Diversity and Dynamics of Agricultural Production Systems in Arid Zones, Biskra, Algeria; 4 Setif University, Applied Microbiology Laboratory, Sétif, Algeria + Jijel University, Faculty of Nature and Life Sciences, Department of Environment and Agronomy Sciences, Jijel, Algeria

**Keywords:** antioxidant, gestational diabetes mellitus, insulin-mimetic, oxidative stress, selenium, supplementation, gestacijski dijabetes melitus, insulin-mimetik, oksidativni stres, selen, suplementacija, antioksidant

## Abstract

**Background:**

In the presence of conflicting advice about the relationship between selenium-type II diabetes-oxidative stress trio, this study aimed to assess the consequences of selenium supplementation on fasting plasma glucose (FPG) level, antioxidant activities of selenodependent and non-selenodependent enzymes, and other markers of oxidative stress studied for the first time during gestational diabetes mellitus (GDM).

**Methods:**

This research was carried out among 180 pregnant Algerian women, 60 of whom were in good health, 60 women with GDM did not take supplements, and 60 women with GDM took selenium orally (50 mg/d) for 12 weeks starting from their second trimester of pregnancy. Blood samples were taken in order to assay FPG level and oxidative stress markers.

**Results:**

Selenium supplementation during GDM has demonstrated its hypoglycemic power in the significant decline of FPG level, and its antioxidant properties in the significant reinforcement of antioxidant activities of erythrocyte selenodependent enzymes (glutathione peroxidase and glutathione reductase), the significant increase in erythrocyte catalase and superoxide dismutase activities simultaneously with the highest decrease in erythrocyte and plasma malondialdehyde levels. This decrease was only significant for plasma carbonyl proteins, which was not the case for erythrocyte carbonyl proteins.

**Conclusions:**

The recourse to selenium supplementation by seleno-deficient pregnant women with GDM is beneficial for maternal health. This micronutrient exploits its antioxidant and insulin-mimetic properties in the maintenance of blood glucose homeostasis and the fight against oxidative stress, and consequently, its supplementation delays the occurrence of GDM complications.

## Abstract (sr)

**Uvod:**

Svesni različitih saveta koji se daju u vezi sa međusobnim odnosom selena, dijabetesa tipa II i oksidativnog stresa, ova studija je imala za cilj da oceni uticaj suplementacije selena na nivo glukoze u plazmi u stanju gladovanja (FPG), antioksidantne aktivnosti enzima koji zavise i koji ne zavise od selena, kao i na druge markere oksidativnog stresa koji su proučavani po prvi put tokom gestacijskog dijabetesa melitusa (GDM).

**Metode:**

Ovo istraživanje je sprovedeno na uzorku od 180 trudnica iz Alžira, od kojih je 60 bilo dobrog zdravlja, 60 žena sa GDM koje nisu uzimale suplemente, i 60 žena sa GDM-om sa oralnom suplementacijom selena (50 mg/d) u periodu od 12 nedelja počev i od drugog trimestra. Nivo FPG i markeri oksidativnog stresa su određeni iz krvi.

**Rezultati:**

Suplementacija selena tokom GDM-a je pokazala svoju hipoglikemijsku snagu značajnim padom nivoa FPG, i njegova antioksidativna svojstva značajnim pojačanjem anti oksidativnih aktivnosti eritrocitnih seleno-zavisnih enzima (glutation peroksidaze i glutation reduktaze), značajnim povećanjem aktivnosti eritrocitne katalaze i superoksid dismutaze istovremeno sa visokim smanjenjem nivoa eritrocita i malondialdehida u plazmi. Ovo smanjenje je bilo značajno samo za karbonilne proteine u plazmi, to nije bio slučaj za karbonilne proteine eritrocita.

**Zaključak:**

Suplementacija selena kod trudnica sa deficitom selena koje imaju GDM je korisna za zdravlje majki. Ovaj mikronutrijent koristi svoje antioksidativne i insulin-mimetičke osobine u održavanju homeostaze glukoze u krvi i borbi protiv oksidativnog stresa, a posledično njegova suplementacija smanjuje pojavu komplikacija u vezi sa GDM-om.

## Introduction

GDM is one of the most common forms of highrisk pregnancies that are in alarming spread [1]. It is a glucose tolerance disorder that occurs for the first time during pregnancy [2], most often during the second trimester of amenorrhea, and disappears just after childbirth [3]. GDM settles in the maternal organism as a result of the lack of control over insulin resistance state associated with pregnancy [3]. This situation is accentuated by the increase in oxidative stress, due to the overproduction of reactive oxygen species (ROS) during pregnancy [4]. In fact, oxidative stress creates a vicious cycle with GDM, one of which promotes the onset of the other [4]. Oxidative stress contributes to the reduction of liver, fat and muscle tissues sensitivity to the insulin action in a mother's body, leading to elevated glucose intolerance [5]. Consequently, the engendered hyperglycemic state, in turn, promotes the production of ROS through various mechanisms such as protein glycation, polyol pathway, NADPH oxidase pathway and glucose autoxidation, which aggravates the situation [6]. Selenium (Se) is an essential trace element for the proper functioning of the body, known for its powerful antioxidant properties [7], which contribute to the maintenance of oxidant/antioxidant balance and the protection of noble molecules (proteins, DNA, carbohydrates and lipids) from radical attacks [8].

The relationship between Se-type II diabetesoxidative stress trio has recently been deepened, with the reported results which have opened a divergence of opinion [8]. Some authors consider Se as a diabetogenic trace element, its dietary supplementation does not contribute to the overexpression of antioxidant seleno-enzymes [9], but it promotes obesity, exacerbation of insulin resistance state and excessive generation of ROS [8] [9] [10], while others have reported that Se has an insulin-mimetic effect, its nutritional supplementation improves insulin signal transmission, decreases oxidative stress, glucose intolerance and delays the onset of diabetes complications [11] [12]. In the presence of these contradictory opinions on Se intervention pathways in carbohydrate metabolism and its influence on oxidant/antioxidant status during type II diabetes, our study has gone into the same subject with an original aspect through the use of a different dose of Se supplementation among pregnant Algerian women with GDM, whose purpose was to define its influence on FPG level during this temporary diabetes and its impact on antioxidant activities of erythrocyte selenodependent enzymes (glutathione peroxidase (GPx) and glutathione reductase (GRase)) and non-selenodependent enzymes (superoxide dismutase (SOD) and catalase (CAT)), and the status of plasma and erythrocyte carbonyl proteins (PC) and malondialdehyde (MDA).

## Materials and Methods

### Recruitment of participants

This research work is a randomized controlled study, carried out among 180 pregnant Algerian women with monofetal pregnancy according to the ethical guidelines of 1964 Helsinki declaration and its later amendments, with approval letters obtained from the direction of LBNT. These women followed the progress of their pregnancy at the obstetrics and gynecology service of Zammit Surgical Clinic in El Eulma-Setif, Algeria. The purpose of our study was very well explained to participants, whose written consent was previously obtained, respecting their anonymity and confidentiality.

Participants were recruited during ten months (January 09 - October 09 2018) after a gynecological obstetric examination, an individual interview and the use of their medical records in order to explore their physical characteristics and some predictive factors of GDM. They were divided into three groups:

The first group (60 healthy pregnant women): they had no pathology or complication associated with their pregnancy. Two blood samples were taken from these women, the first between the 24th and 28th gestational week and the second after 12 weeks.

The second and the third group were each composed of 60 pregnant women with GDM subjected to insulin therapy (1 to 4 insulin injections per day according to the capillary blood glucose results), moderate physical activity (3 times per week) and special dietary regime. The latter was based on the minimization of daily dietary intake of sugar (35 to 40% of total daily caloric intake distributed over 3 meals and 2 to 3 snacks) and lipids (especially food high in saturated and Trans-fatty acids), and the increase in daily intakes of protein and dietary fibre. Good hydration and food diversification were recommended to satisfy the foeto-maternal needs for minerals and vitamins [3].

These women were recruited according to the criteria of the one-step method for GDM screening established by WHO (2013) [3], with no other pathology or complication associated with pregnancy. Only one blood sample was taken from the second group (non-supplemented women with GDM) between the 37th and 40th gestational week. Patients in the third group (Se-supplemented women with GDM) whose gestational age was 24 to 28 weeks, were orogastrically supplemented with 50 μg/d Se in the form of L-Selenomethionine capsules (Laboratory Nutrixeal® , Meylan, France) for 12 weeks. Two blood samples were taken from patients in this group, the first of which was collected before Se supplementation and the second after 12 weeks.

All women involved in this study were systematically supplemented with 60 mg/d iron and 400 mg/d vitamin B_9_ during pregnancy as recommended by WHO (2016) [13], and they did not receive any other nutritional supplement one month before and during pregnancy. Women who needed other micronutrient supplementation were excluded from this study to avoid their influence on the results.

To monitor the side effects of Se supplementation and to prevent participants from GDM complications, Se-supplemented patients had a medical consultation with a diabetologist every 15 days and with an obstetrician-gynecologist every 30 days [14].

It should be noted that women with unrecognized diabetes, type I or type II diabetes were not involved in this study; the subjects were women who developed GDM and they were all asked to keep their insulin therapy and hygienic-dietary measures.

### Assay of Se, FPG levels and oxidative stress markers

Blood samples were taken on heparinized tubes. After centrifugation, recovered plasma was destined for assaying: Se level by Agilent 240Z AA graphite furnace atomic absorption spectrophotometer with Zeeman-effect (Agilent Technologies, Santa Clara, USA) using 196 nm wavelength, and FPG level according to the enzymatic method described in the commercial Biomaghreb Kit (Biomaghreb Laboratory, Tunis, Tunisia).

The obtained blood pellet was intended to prepare erythrocyte lysate in order to be able to assay antioxidant status markers by UV-Visible spectrophotometry: Enzymatic activity of CAT by the use of Aebi's method [15], and enzymatic activities of SOD, GPx and GRase by the use of commercial Randox kits (Randox Laboratories, Crumlin, United Kingdom).

Plasma and erythrocyte PC and MDA (oxidant status markers) assaying was carried out according to the method published by Draper and Hadley in 1990 [16], and Levin and his collaborators in 1990 [17] respectively.

### Statistical analysis

Statistical processing of data was carried out using SPSS statistics software version 25. Results are expressed as means ± standard deviation for α=5%. Comparing means of the three groups was carried out by ANOVA parametric test, and in order to find out exactly where the difference is Scheffé test was applied in the presence of homogeneity and Tamhane test in the absence of homogeneity. Student's T-test for two paired samples was used to compare means of the same group, and Student's T-test for two unpaired samples was used to compare the means of two different groups. The difference is significant if P<0.05.

## Results

Pregnant women with GDM included in this study were overweight, and were more exposed to personal history of GDM and macrosomia, and firstdegree family history of type II diabetes compared to healthy control pregnant women (Table 1).

**Table 1:** General characteristics of healthy pregnant women, non-supplemented and Se-supplemented pregnant women with GDM Data are expressed as means±standard deviation or absolute number (percentage); Statistical significance is identified if P<0.05; P^a^: P-value for healthy pregnant woman group; P^b^: P-value for Se-supplemented women with GDM group; P^c^: P-value for the 03 groups; GDM: Gestational diabetes mellitus, BMI: Body mass index, SBP: Systolic blood pressure; DBP: Diastolic blood pressure.

Characteristics		Healthy pregnant women group (n=60)		*P* ^a^ value	Se-supplemented GDM group (n=60)		*p^b^* value	Non-supple- mented GDM group (n=60)	*p^c^* value
		W0	W12		W0	W12			
Maternal age (y)		28.28±4.19			31.63±4.12			32.77±3.89	0.000
Gestational age (W)		25.87±1.28			26.58±0.97			38.82±0.63	0.000
BMI (kg/m^2^)		25.61±0.76	26.83±0.75	0.000	29.11±0.69	30.28±0.69	0.000	30.33±0.75	0.000
SBP (mm Hg)		108.83±3.66	111.35±4.09	0.000	110.53±2.92	113.87±2.61	0.000	113.77±2.45	0.000
DBP (mm Hg)		68.27±2.58	71.35±2.77	0.000	67.52±2.13	68.60±1.78	0.000	70.32±1.63	0.000
Personal history of GDM % (n/60)	Yes	0% (0/60)			5% (3/60)			8.30% (5/60)	0.000
	No	100% (60/60)			95% (57/60)			91.70% (55/60)	0.000
Personal history with macrosomia % (n/60)	Yes	3.30% (2/60)			10% (6/60)			10% (6/60)	0.000
	No	96.70% (58/60)			90% (54/60)			90% (54/60)	0.000
First-degree family history of type II diabetes % (n/60)	Yes	8.30% (5/60)			36.70% (22/60)			40% (24/60)	0.000
	No	91.70% (55/60)			63.30% (51/60)			60% (48/60)	0.000

Before Se supplementation, pregnant women with GDM had significantly lower plasma Se level associated with fasting hyperglycemia, and were more vulnerable to oxidative stress increase, whose levels of plasma and erythrocyte MDA and PC were significantly higher, and levels of all studied markers of antioxidant defence (erythrocyte enzymatic activities of GPx, GRase, CAT and SOD) were reduced compared to healthy women group with normoglycemic pregnancies (Table 2).

**Table 2:** Se, FPG levels and oxidant/antioxidant status in healthy pregnant women and Se supplemented women with GDM groups at study baseline and after 12 weeks of intervention Data are expressed as means ± standard deviation. Statistical significance is identified if P<0.05; P^a^: P-value for the same group; P^b^: P-value from W0 of the two groups; GDM: Gestational diabetes mellitus, Se: Selenium; FPG: Fasting plasma glucose; GPx: Glutathione peroxidase; GRase: Glutathione reductase; CAT: Catalase; SOD: Superoxide dismutase; MDA: Malon dial-dehyde; PC: Carbonyl protein.

Parameters	Healthy pregnant women group (n=60)			Se-supplemented GDM group (n=60)			P^b^ value
	W0	W12	P^a^ value	W0	W12	P^a^ value	
Se (μg/L)	89.68±13.91	85.57±13.62	0.000	67.88±13.71	81.89±13.45	0.000	0.000
FPG (g/L)	0.80±0.06	0.84±0.04	0.000	1.15±0.07	0.97±0.07	0.000	0.000
Markers of antioxidant status							
GPx (U/g Hb)	48.37±8.26	46.60±8.14	0.000	33.75±8.48	42.83±8.48	0.000	0.000
GRase (U/g Hb)	9.48±2.12	8.31±2.05	0.000	5.80±1.88	7.96±1.89	0.000	0.000
CAT (U/g Hb)	77.70±11.68	75.04±11.19	0.000	52.02±11.46	55.55±11.43	0.000	0.000
SOD (U/g Hb)	987.36±108.34	951.49±115.59	0.009	814.70±113.69	821.91±113.19	0.000	0.000
Markers of oxidant status							
Plasma MDA (μmol/L)	1.97±0.50	2.67±0.58	0.000	3.56±0.55	2.78±0.56	0.000	0.000
Erythrocyte MDA (μmol/L)	4.22±0.94	5.04±0.87	0.000	6.72±1.00	5.63±1.00	0.000	0.000
Plasma PC (μmol/L)	2.59±0.65	3.43±0.62	0.000	3.83±0.66	2.81±0.65	0.000	0.000
Erythrocyte PC (μmol/L)	5.06±0.87	5.98±0.94	0.000	7.44±0.96	6.70±2.17	0.005	0.000

After 12 weeks, Se supplementation in patients with GDM resulted in a tangible improvement in their carbohydrate metabolism, reflected in the highly significant decrease of their FPG level compared to their baseline status and compared to that of non-supplemented GDM group (Table 3).

**Table 3:** Se, FPG levels and oxidant/antioxidant status in healthy pregnant women, Se-supplemented and non-supplemented women with GDM groups after 12 weeks of intervention Data are expressed as means ± standard deviation. Statistical significance is identified if P<0.05; P^a^: P-value for healthy pregnant women and Se-supplemented women with GDM groups; P^b^: P-value for Se-supplemented and non-supplemented women with GDM groups; GDM: Gestational diabetes mellitus; Se: Selenium; FPG: Fasting plasma glucose; GPx: Glutathione peroxidase; GRase: Glutathione reductase; CAT: Catalase; SOD: Superoxide dismutase; MDA: Malondialdehyde; PC: Carbonyl protein

Parameters	Healthy pregnant women group (n=60)	Se-supplemented GDM group (n=60)	P^a^ value	Non-supplemented GDM group (n=60)	P^b^ value
Se (mg/L)	85.57±13.62	81.89±13.45	0.346	62.97±14.10	0.000
FPG (g/L)	0.84±0.04	0.97±0.07	0.000	1.16±0.06	0.000
Markers of antioxidant status					
GPx (U/g Hb)	46.60±8.14	42.83±8.48	0.050	31.39±8.51	0.000
GRase (U/g Hb)	8.31±2.05	7.96±1.89	0.701	4.07±1.44	0.000
CAT (U/g Hb)	75.04±11.19	55.55±11.43	0.000	49.16±10.98	0.009
SOD (U/g Hb)	951.49±115.59	821.91±113.19	0.000	780.43±114.07	0.142
Markers of oxidant status					
Plasma MDA (μmol/L)	2.67±0.58	2.78±0.56	0.524	4.67±0.45	0.000
Erythrocyte MDA (μmol/L)	5.04±0.87	5.63±1.00	0.004	8.29±0.99	0.000
Plasma PC (μmol/L)	3.43±0.62	2.81±0.65	0.000	4.97±0.68	0.000
Erythrocyte PC (μmol/L)	5.98±0.94	6.70±2.17	0.060	8.83±0.95	0.000

This supplementation had a positive impact on the rearrangement of oxidant/antioxidant balance, to the extent that there was no significant difference in plasma Se level, erythrocyte PC level, plasma MDA level and antioxidant activities of GPx and GRase between the Se-supplemented GDM group and the healthy pregnant women group (Table 3).

Although Se supplementation in pregnant women with GDM led to a highly significant increase in SOD and CAT enzymatic activities compared to their baseline status, this increase was only significant for CAT activity compared to the group of non-supplemented diabetic patients. The latter had significant fasting hyperglycemia, severe deterioration in Se status and a significant increase in oxidative stress compared to the Se-supplemented GDM group and healthy pregnant women group (Table 3).

## Discussion

Until today, there is no consensus on Se dietary supplementation impact during type II diabetes, which some studies consider as a micronutrient that favours diabetes occurrence and decreases tissues sensitivity to insulin hormone, while others consider it as an insulin-mimetic trace element which improves sugar metabolism. During our bibliographic research, we only found one study that addressed the effect of dietary supplementation with Se (200 μg/d for 6 weeks) on FPG and 4 markers of oxidative stress (GSH, plasma MDA, nitric oxide and total antioxidant capacity of plasma) in pregnancies complicated by GDM [18]. Our study constitutes a new scientific approach, which has focused on this topic with a larger sample of pregnant Algerian women with GDM (n=60 vs n=35). These patients took a lower dose of Se (50 μg/d vs 200 μg/d) for a longer period (12 weeks vs 6 weeks), with an aim to investigate its influence on carbohydrate metabolism and to determine for the first time its impact on erythrocyte antioxidant activities of GPx, GRase (Se is their cofactor), SOD (zinc is its cofactor), and CAT (iron is its cofactor), as well as its effect on plasma and erythrocyte oxidant status markers (MAD and PC).

During the period of our scientific research, oral nutritional supplementation with Se did not cause any side effects. The same observation was reported among Se-supplemented British women with normoglycemic pregnancies (60 μg/d) from their 12th gestational week to delivery [19].

Statistical analysis in the present study revealed a highly significant increase in plasma Se level of Sesupplemented patients, associated with a substantial decrease in their FPG level. These results corresponded to those reported by Asemi Z and his collaborators [18] and showed the effectiveness of using a lower dose of Se nutritional supplement in the regulation of sugar metabolism and the enhancement of maternal tissues sensitivity to the hypoglycemic action of insulin during GDM in seleno-deficient women. These benefits resemble those observed after the use of chromium supplementation as another trace element during this type of high-risk pregnancies [20], the time when the recourse to iron supplementation was positively associated with significant elevation in FPG level [21]. The hypoglycemic power of Se and its ability to delay diabetes complications are thus exalted during type II diabetes, but exclusively in deficient or sub-deficient people in this micronutrient [22]. However, a French study reported that the anti-diabetogenic properties of Se occur only in men and reduce the risk that they develop type II diabetes to 50% [23]. The beneficial effect of this micronutrient supplementation (200 μg/d for 06 weeks) in the reduction of exacerbated state of insulin resistance has been proven in other diseases such as polycystic ovary syndrome [24] and central obesity [25]. In contrast, other studies have advised against the use of Se supplementation, considering it as a promoting factor of type II diabetes in healthy women [23] and mice [8], which leads to the worsening of sugar metabolism disorders [24], especially in the Caucasian race [12], and in seleniferous zones [9]. Se is a trace element involved in the regulation of energy metabolism at several levels [11]. It seems that Se exploits its antioxidant properties in the reduction of oxidative stress intensity which is an important factor promoting the insulin resistance amplification [26], and it is involved in gluconeogenesis pathways inactivation in the presence of excess free glucose by promoting its uptake for the synthesis of adipose tissues and hepatic glycogen, through the induction of peroxisome proliferator-activated receptors gamma overexpression, and the inactivation of protein tyrosine phosphatases (PTPs) that impede insulin signal transduction and therefore the improvement of insulin sensitivity [11] [26].

The results of this research work showed that in the second trimester of pregnancy, antioxidant defence of healthy pregnant women was significantly more potent than that of pregnant women with GDM, but after 12 weeks, healthy pregnant women had a significant increase in their oxidative stress but in a manner that still could be controlled. This is due to the progress of the pregnancy, whose ROS are notably generated by the placenta to play an important role in cell signal transduction [27]. However, oxidative stress was significantly more severe during the third trimester of pregnancy in non-supplemented pregnant women with GDM, whose excess ROS threaten maternal health by disrupting the biological function of lipids, carbohydrates, DNA and proteins [27].

The results of this study have demonstrated the benefits of Se supplementation in the reduction of protein oxidation and lipid peroxidation. Its efficacy in the reduction of plasma MDA level is also noted in Iranian patients developing GDM [18] and in patients suffering from renal failure after their supplementation (200 μg/day Se for three months) [28]. The collected data from research work carried out on animal models expressed that Se supplementation induced a significant decrease in hepatic MDA level in mice with psoriasis [29], and cerebral MDA level in male rats subjected to intense physical activity [30].

The ability of this micronutrient to improve enzymatic activities of erythrocyte GPx and GRase in synchronisation with the decline of oxidant status markers was proven during experimental diabetes among Se supplemented Wistar rats (02 μmol/kg/d for 12 weeks) [31]. It appears that the use of Se supplementation during GDM is more effective than that of probiotics for enhancing enzymatic activities of erythrocyte GPx, GRase, and even SOD [32]. CAT and SOD antioxidant activities were significantly recovered in Se-supplemented patients in this study. These positive effects were indicated for hepatic CAT and SOD of intoxicated rats with cadmium after their treatment with Se and the extract of Aerva Monsoniae [33].

In conclusion, pregnant Algerian women with GDM included in this study, were seleno-deficient, their supplementation with Se (50 μg/d for 12 weeks) from their second trimester of pregnancy, resulted in the best manifestation of its insulin-mimetic and antioxidant properties that led to an improvement in carbohydrate metabolism expressed by significant decrease in FPG level, a strengthening in selenodependent antioxidant defense, a recovery of SOD and CAT activities and a significant decrease in plasma and erythrocyte MDA levels and plasma PC level. Due to the financial obstacles and time constraints, we could not look deeper into the influence of Se supplementation on the redox status of placenta and newborns, mode of childbirth, neonatal glycemia, maternal urinary tract infections, occurrence of macro somia and long-term complications of GDM in mothers and their progeny, this is why several studies are needed to enrich data and deepen knowledge on this research topic with other Se doses, in other regions and with other ethnic groups.

## Financial disclosure

This study was supported by the Laboratory of Nuclear Biotechnology and Technology (LNBT) of Sidi Thabet technopark and University of Tunis El Manar. The authors have no financial relationships relevant to this article to disclose.

## Ethical approval

All protocols in this scientific research were in accordance with the 1964 Helsinki declaration and its later amendments or comparable ethical standards and were approved by the direction of LNBT of Sidi Thabet technopark.

## Author contributions

Hadjer Saifi conducted the study, collected the data, contributed to the interpretation of the findings and wrote the first draft of the manuscript. Mouldi Saidi and Yassine Mabrouk contributed to the conception, design, and supervision of the study. Messaouda Benabdelkader and Rayane Saifi carried out the data analysis, contributed to the interpretation of findings and significantly improved the manuscript.

## Conflict of interest statement

The authors stated that they had no conflicts of interest regarding the publication of this article.
